# Reproductive Ecology and Severe Pollen Limitation in the Polychromic Tundra Plant, *Parrya nudicaulis* (Brassicaceae)

**DOI:** 10.1371/journal.pone.0032790

**Published:** 2012-03-12

**Authors:** Justin R. Fulkerson, Justen B. Whittall, Matthew L. Carlson

**Affiliations:** 1 Biological Sciences Department, University of Alaska Anchorage, Anchorage, Alaska, United States of America; 2 Department of Biology, Santa Clara University, Santa Clara, California, United States of America; 3 Alaska Natural Heritage Program, University of Alaska Anchorage, Anchorage, Alaska, United States of America; Lakehead University, Canada

## Abstract

Pollen limitation is predicted to be particularly severe in tundra habitats. Numerous reproductive patterns associated with alpine and arctic species, particularly mechanisms associated with reproductive assurance, are suggested to be driven by high levels of pollen limitation. We studied the reproductive ecology of *Parrya nudicaulis*, a species with relatively large sexual reproductive investment and a wide range of floral pigmentation, in tundra habitats in interior montane Alaska to estimate the degree of pollen limitation. The plants are self-compatible and strongly protandrous, setting almost no seed in the absence of pollinators. Supplemental hand pollinations within pollinator exclusion cages indicated no cage effect on seed production. Floral visitation rates were low in both years of study and particularly infrequent in 2010. A diversity of insects visited *P. nudicaulis*, though syrphid and muscid flies composed the majority of all visits. Pollen-ovule ratios and levels of heterozygosity are consistent with a mixed mating system. Pollen limitation was severe; hand pollinations increased seed production per plant five-fold. Seed-to-ovule ratios remained low following hand pollinations, indicating resource limitation is likely to also be responsible for curtailing seed set. We suggest that pollen limitation in *P. nudicaulis* may be the result of selection favoring an overproduction of ovules as a bet-hedging strategy in this environmental context of highly variable pollen receipt.

## Introduction

Pollen limitation, or the reduction in reproductive success due to an inadequate supply of pollen [1]–[3], is predicted to be strongest in environments where pollinators are low in abundance or where pollinator services are unreliable [4]–[6]. Nowhere else is pollinator service more unreliable and persistently low than in arctic and alpine environments. High latitude and alpine plants that depend on pollinator services are most likely to be pollen limited because insect pollinators are lower in diversity and abundance in these habitats [7]–[12]; competition among plants for pollinator services should therefore also be elevated in arctic and alpine habitats [7]. Additionally, the climate in tundra habitats is typically cold and windy, curtailing flying insect foraging time [7], [13], [14]. Furthermore, the pollinating insect assembly in these habitats is skewed toward flies (Diptera) [9], [13], [15]–[17], which are generally considered inefficient pollinators compared to bees [18], [19], but are vital for reproduction in some arctic plant species [20].

Severe levels of pollen limitation can have significant evolutionary consequences. Species with mechanisms for reproductive assurance are expected to persist in conditions of chronically low pollinator visitation [4], [21], [22]. Pollen limitation has been proposed to be the cause for the high frequency of selfing mechanisms and asexual reproduction among arctic and alpine taxa [12], [23]. Indeed, pollinator-dependent reproductive systems are considered to be extremely rare in the higher latitudes due to the unreliability of pollinator services [24]–[29]. Furthermore, tundra environments are considered to be dominantly consisting of wind-pollinated, highly self-fertilizing, vegetatively reproducing, or apomictic plants [24]–[28], [30]. The emphasis on reproductive assurance in the tundra flora initially led to the notion that pollination service may be irrelevant [31]–[33]. A surprising number of arctic species, however, are reliant on pollinators for sexual reproduction [8], [20], [29], [34], [35].

Aside from pollen availability, soil nutrient resources, light, climate, water, and resources from previous years can affect seed production [36]–[38], especially among arctic and alpine plants [24], [25], [27], [30], [39], [40]. Haig and Westoby [36] suggested that selection should drive floral traits to an equilibrium, in which the ability to mature seeds is equally constrained by pollen availability and the availability of other resources. Therefore, the addition of pollen should not increase fitness since there should not be available resources for the extra fertilized ovules [4]. Despite these predictions, pollen limitation is common, occurring in 62% to 73% of studies [4], [41]. The frequency of pollen limitation has been suggested to stem from changes in the environment, such as invasive plant introductions [42], habitat fragmentation [43], or a change in the native pollinator assemblage, disrupting the Haig Westoby equilibrium [1], [44], [45]. Alternatively, the widespread observation of pollen limitation may be an evolutionary response where selection favors a greater number of ovules produced than on average are fertilized; individuals with higher numbers of ovules produced throughout the growing season are more likely to capitalize on uncommon pollination events [5], [46]. Literature reviews suggest that this ‘bet-hedging strategy’ is indeed beneficial in stochastic environments where pollinators vary the mating success of individuals, allowing exploitation of occasional large pollen loads [1], [5], [46]. Under this hypothesis, selection is predicted to favor individuals with a larger number of ovules in habitats with inconsistent pollinator visitation rates such as high latitudes and elevations [5].

The dearth of pollinators at higher latitudes has prompted the notion that flowers in these regions are mere vestigial organs [31], [32]. However, the arctic flora harbors many species with large and vibrantly colored flowers that produce nectar and odors apparently for pollinator attraction [7], [8], [47], [48]. An alternative hypothesis is that the investment in rewards and advertisements for pollinators is in fact adaptive and that outcrossing events are important even if they are rare. One such species with relatively large and often vibrantly colored floral displays that occurs in subarctic alpine and arctic regions of North America and Russia is *Parrya nudicaulis* (Brassicaceae). *Parrya nudicaulis* is suggested to be self compatible, but mostly dependent on pollinators for reproduction in the Russian Far-East [34]. This species ranges from white to deep violet in flower color in many populations [49]. Furthermore, as flower color variation is often associated with pollinator selection for specific color morphs [50]–[53], we expect visitation and pollen limitation rates to vary by flower color. Here we evaluate the level of pollen limitation of *P. nudicaulis* across two years in interior Alaska. Specifically, we examined the following questions: 1) are floral visitation rates low and correlated with floral pigmentation? 2) Does pollen limitation occur in *Parrya nudicaulis* and is floral pigmentation related to the level of pollen limitation? Last, 3) does this species have a selfing mating system?

## Materials and Methods

### Ethics

No specific permits were required for the described field studies. The field studies occurred on public lands and were considered casual use by the Bureau of Land Management. The field studies did not involve endangered or protected species.

### Study System

**Figure 1:**
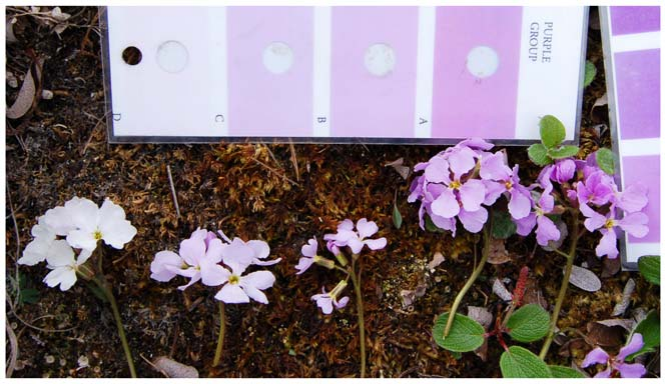
Flower color variation. Flower color is highly variable within this population of *Parrya nudicaulis*. A gradient of violet-purple coloration increases from pure white flowers on the left to dark violet flowers on the right. A Royal Horticultural Society Color Chart serves in the background for comparison.

*Parrya nudicaulis* L. Regel (Brassicaceae) extends from northeast Asia, across Alaska and to the western Canadian Arctic Archipelago [54]. It is found in arctic and alpine tundra regions that typically have moist, sloped, or open sandy habitats. Flowering occurs in late May to mid June in the lower latitudes of Alaska and several weeks later at higher latitudes. This long-lived perennial usually produces a single raceme of 8–14 flowers, which normally persists between 10–14 days with individual flowers senescing after three days. Floral maturation is asynchronous with up to four flowers fully open at a time. Flowers are protandrous; the upper anthers dehisce shortly after the flowers open, followed by the lower two anthers within approximately 12 hours, and the stigma becomes bilobed and receptive during the second day. Corollas are conspicuous with petals averaging 8.98 mm in length and 9.20 mm in width. Flower color of *P. nudicaulis* is variable among individuals in many populations [49]. While the hue is quite consistent, the lightness (or brightness) values range dramatically among individuals creating flowers from pure white to dark violet (Figure 1). All flowers emit a sweet fragrance that is comparable to lilacs. Nectar is secreted at the base of the corolla and less than 4 µL approximately is produced in plants bagged for 24 hours (*personal observation*). *Parrya nudicaulis* is suggested to be preferentially entomophilous for sexual reproduction [35], but it is capable of vegetative propagation via rhizomes [54]. Excavations of several plants have revealed rhizomes to be up to 1 meter in length, yet all branches exhibit the same flower color (*personal observation*).

### Pollinator Observations

The study occurred at Eagle Summit (65° 28′N, 145° 25′W) at 1100 m elevation in the White Mountains of interior Alaska consisting of mesic forb-ericaceous shrub tundra. Video recordings occurred between 16–18 June in 2009 and 2–9 June in 2010. Floral visits were recorded between 10:00–17:00 on randomly selected plants during 30 minute periods when weather permitted (>10°C, light wind and no rain; i.e., conditions when pollinating insect species were observed to be active; also see [14]). Ad hoc observations were made in the morning, evening, night, and under poor weather, but no floral visitors were observed. Video cameras were placed approximately two meters away from the targeted inflorescences. The amount of time where an insect came in contact with either the stigma or anther was defined as a visit and recorded in seconds. Visitation rates were low in both years and we therefore combined observations in our analysis, giving a total of 35 individual floral visitors with 184 open flowers observed. Flower visitation rate was standardized as visits per flower per hour (VFH) for all species combined and was square root transformed to meet normality assumptions for analyses. We used linear regression analysis of flower visitation on flower color (lightness) to determine if the variation in pollinator visitation rates related to flower color.

### Pollination Treatments

A total of 90 experimental plants were randomly selected in each treatment along random transects. To avoid over-sampling of intermediate flower colors, which are most common in this population, we stratified sampling by three main colors categories: white, light violet, and dark violet. Each treatment consisted of 30 individuals of each main color category and Royal Horticultural Society Colour Charts [55] were used to more precisely define flower color. Plants were located at least one meter apart and contained the same number of flowers. In 2009, treatments occurred 14–17 June during mid/late-season of *P. nudicaulis* flowering time. The 2010 treatments occurred at the beginning of *P. nudicaulis* flowering season from 31 May to 7 June. Manipulated flowers were marked with a small amount of paint at the base of the pedicel. Infructescences were collected at the end of July, prior to seed dehiscence, and fitness was quantified as seeds/fruit and seeds/plant, as they are the most appropriate measures of response for pollen limitation studies [56]. Seed set was square root transformed to meet the assumptions of normality. To test if pollinators are required for seed set in *Parrya nudicaulis*, we compared seed production between plants open to pollination (−HP −CAGE) and plants with pollinator exclusion cages (−HP +CAGE). A cylindrical cage was constructed from poultry wire and fine bridal veil netting secured around the frame to keep pollinators from visiting the entire inflorescence [57]. Although bridal veil netting is a popular method for pollinator exclusion, it can possibly alter the microclimate of the flowers [57]. We controlled for cage effects by including a separate treatment of hand-pollinated plants (+HP −CAGE) paired with a treatment consisting of both hand-pollination and an exclusion cage (+HP +CAGE). To test if pollinators were required for seed set we used a *t*-test to see if the open pollination treatment was significantly different than the cage treatment. To determine if there were cage effects, we used a *t*-test determine if the cage with hand pollination was significantly different than the hand pollination treatment alone. PASW Statistics 18 was used for all statistical analysis in this paper [58].

We compared seed production between a supplemental hand pollination treatment and a control treatment that was open to pollination to estimate the degree of pollen limitation. Anthers of at least ten randomly selected individuals of different flower colors that were greater than 30 meters away from the transect were collected and placed in a vial. The mixed pollen vial was refreshed every hour to maintain pollen viability. A metal probe was used to cover an estimated 50% of the stigma surface with outcrossed and self pollen. Every flower was hand-pollinated every day, until there were signs of flower senescence to ensure that stigma receptivity was not missed and differential ovule maturation would not affect seed set. Supplemental pollen added to the entire inflorescence reduces the chance of resource allocation interfering with the detection of pollen limitation and is therefore regarded better than a partial inflorescence supplementation [4], [37], [56]. Because the flowers had already opened upon our arrival in 2009, we were unable to apply pollen to the entire inflorescence in that year. We used the data for determining if cage effects were present since both hand pollination (+HP −CAGE) and cage with hand pollination treatments (+HP +CAGE) were partial inflorescence supplemented. We used only the 2010 supplemental hand pollination data for pollen limitation analysis since it was applied to the entire inflorescence. To test whether pollen limitation occurred we used a one-tailed *t*-test to see if the supplemental hand pollinated treatment produced significantly more seed than the open pollination treatment. We used linear regression analysis of seed set on flower color (lightness) to determine if the magnitude of pollen limitation was related to flower color.

### Quantification of Flower Color

The variation of flower color in *P. nudicaulis* in this population is continuous. A Royal Horticultural Society Colour Chart [55] was laminated and used to quantify color between plants at the time of anthesis. Using this chart, however, limits the factor of ‘color’ to categorical data. To determine lightness values of the color chips, the laminated RHS Colour Chart was scanned at a setting of ‘600 dpi full color glossy’ on a RICOH Aficio MP 4000 scanner and saved as a ‘.tiff’ file. The image was converted to CIE-L*u*v* color values using Colour Transform application in ImageJ [59]. Lightness or L* values were averaged for the RHS color chip by taking 10 random points along two transects of each color chip. Although the same method to quantify color using HSB values (hue, saturation, and brightness) has been used to differentiate between varying degrees of brightness in petal color [60], HSB is a human specific color space [61]. Color quantification is well studied among the animal scientific community who prefers to use a well tested color space system such as RGB (red, green, blue) or one developed by the International Commission on Illumination Laboratory (Commission Internationale de l'Eclairage *known as* CIE colour space) [61]. The CIE L* values used are a measure of lightness where the L* values range from 0 to 100, where ‘0’ is black or ‘near-black’ and ‘100’ is white or ‘near-white’ and are differentiated within the RHS Colour Chart as effectively as light spectroscopy [62]. Since *P. nudicaulis* petals fall within a narrow range of purple-violet of the RHS Colour Chart, and L* is highly correlated with anthocyanin concentration (Whittall et al. unpublished data), lightness values (L*) are a good reflection of the range of flower color observed in *P. nudicaulis*.

Flower color does not correlate with flower size and therefore does not confound our ‘flower color’ variable (Pearson Correlation Coefficients of color on: petal length *r*_(172)_ = 0.069, *p* = 0.364; petal width *r*_(172)_ = 0.045, *p* = 0.560; corolla depth *r*_(172)_ = 0.016, *p* = 0.837; height tallest anther *r*_(170)_ = 0.062, *p* = 0.424). Overall mean petal length was 8.84 mm±0.09 SE, mean petal width was 9.25 mm±0.09 SE, mean corolla depth was 9.40 mm±0.07 SE, and mean height of the tallest anther was 9.40±0.07 SE. A comparison of 12 white and 10 dark flowered individuals from a nearby population produced similar amounts of pollen per flower (unequal variances, t-test, *p* = 0.36), suggesting that flower color is not correlated with pollen rewards.

### Pollen and Ovule Counts

We randomly chose 98 flowers from the 180 individuals of the 2010 year treatments for ovule counts. Collected flowers were of the same flowering stage and location on the inflorescence. Flowers were stored in denatured alcohol and ovules were later counted at 10× magnification. Pollen had already dehisced at the time these flowers were collected, therefore, a separate nearby random transect was used to collect all six undehisced anthers from 21 individuals of the same flowering stage in denatured alcohol. Anthers were transferred to a vial containing DI water and sonicated for one minute until the pollen was released from the anther sacs [63], [64]. One drop of diluted liquid detergent was placed in the vial and vortexed to suspend and equally distribute pollen grains in the vial. Pollen grains were counted under a microscope and total pollen per flower was estimated from aliquoted pollen solutions and anther number [65]. Although the pollen and ovule counts are not from the same flower or individual, this method provides reliable population-level estimates of pollen to ovule ratio. Pollen to ovule ratio (P/O-ratio) of *P. nudicaulis* was compared to the outcrossing index given by Cruden [65]. Seed to ovule ratio (S/O-ratio) of *P. nudicaulis* was compared to S/O-ratio plant life history index given by Wiens [66].

## Results

### Flower visitation rates

**Figure 2:**
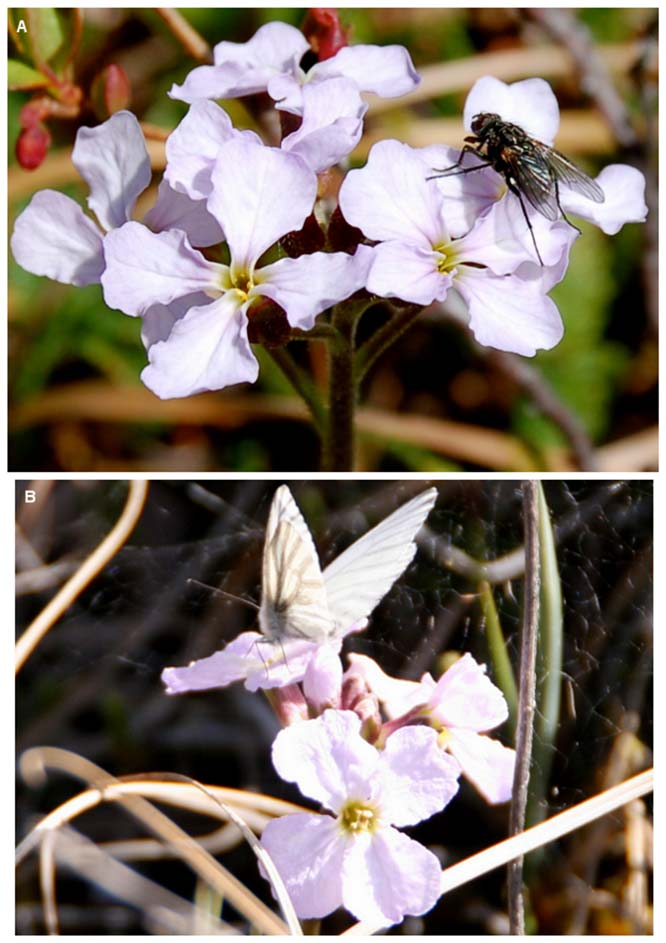
Floral visitors. 2A. Muscid fly and 2B. *Pieris angelika* visiting *Parrya nudicaulis*.

The weather during flowering time in 2009 was warm and favorable for insect flight unlike the 2010 season where it was marked with numerous thunderstorms and rain showers. Of the 52 flower hours observed in 2009, the mean visitation rate was 0.582 VFH. The mean visitation rate was 0.14 VFH for 40 flower hours of observation in 2010. Flower color did not explain a significant amount of variation in visitation rate of flowers (Linear Regression: *r^2^* = 0.023, *F*_(1,34)_ = 0.017, *p* = .897). Flower visits were dominated by dipterans. The most frequently observed flower visitors were syrphid flies representing 59% of the visits followed by muscid flies representing 23% of the visits. *Rhamphomyia* spp. (Diptera: Empidae) were observed less frequently with 7% of the visits. On occasion we have observed *Pieris angelika* (Lepidoptera) and *Boloria* spp. (Lepidoptera) to visit *P. nudicaulis* at this site but they were not captured by these video recordings. Photographs of common flower visitors are shown in Figure 2.

### Does pollen limitation occur in *Parrya nudicaulis*?

**Figure 3:**
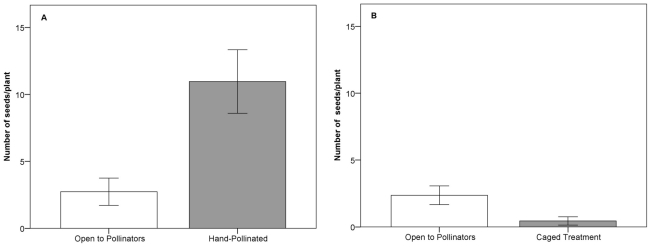
Seeds per plant relative to pollination treatments. 3A. Mean number of seeds per plant in control open to pollinators and supplemental hand pollination treatments in 2010. 3B. Mean number of seeds per plant in open to pollinator control and pollinator excluded (“caged”) treatments from 2009. Error bars indicate 95% CI.

Supplemental hand pollination (+HP −CAGE) significantly increased the number of seeds per plant in *P. nudicaulis* by nearly 500% (*t* test, equal variance; *t* = −4.74, df = 105, *p*<0.001). Hand pollinated plants in 2010 produced a mean 10.96±1.19 SE seeds/plant compared to the control treatment (−HP −CAGE) that produced only 2.74±0.51 SE seeds/plant (Figure 3). There was also a similar significant difference detected for seeds per fruit (*t* test, equal variance; *t* = −6.59, df = 105, *p*<0.001), where hand pollinated flowers yielded 2.02±0.22 SE seeds/fruit and the controls yielded just 0.33±0.06 SE seeds/fruit. Flower color did not explain a significant amount of variation in pollen limitation (Linear Regression: *r^2^* = 0.01, *F*_(1,83)_ = 1.17, *p* = 0.28) or seeds per fruit (Linear Regression: *r^2^* = 0.012, *F*_(1,83)_ = 1.03, *p* = 0.312). There was no significant difference in seed per plant in open pollinated plants between 2009 and 2010 (*t* test, unequal variances; *t* = .433, df = 168, *p* = .666).

### Are pollinators required for seed set in *Parrya nudicaulis*?

*Parrya nudicaulis* is capable of producing a minor amount of seed in the absence of pollinators; however seed production is approximately five times greater in the presence of pollinators (Figure 3). Plants that were excluded from pollinator visitation (−HP +CAGE) produced a mean of 0.45±0.15 SE seed/plant compared to plants open to pollinator visitation (−HP −CAGE) produced 2.37±0.35 SE seeds/plant, a highly significant difference (*t* test, equal variance; *t* = 6.67, df = 172, *p*<0.0001). A significant response was also seen in seeds per fruit where the mean was 0.09±0.02 SE seeds/fruit in the pollinator exclusion treatment and 0.61±0.08 SE seed/fruit in plants open to pollinators (*t* test, equal variance; *t* = 6.24, df = 172, *p*<0.0001). There was no difference in fitness between the cage with hand pollination treatment (+HP +CAGE) and the hand pollinated only treatment (+HP −CAGE) for both seeds/plant (*t* test, unequal variance; *t* = −0.79, df = 97, *p* = 0.432) and seeds/fruit (*t* test, unequal variance; *t* = −1.03, df = 97, *p* = 0.302) indicating that the cage alone did not influence seed set.

### Does *Parrya nudicaulis* have a selfing mating system?

Flowers of *P. nudicaulis* have a mean of 11.60±0.29 SE ovules and 14,690±1,180 SE pollen grains yielding a P/O-ratio of 1,266, consistent with a facultative outcrossing mating system rather than a selfing mating system [65]. Facultative and obligate selfing species generally have P/O-ratios under 200 [65]. The percentage of ovules developing into seeds is quite low in both control and hand pollinated treatments. The seed/ovule (S/O)-ratio was 0.052 for open pollinated controls in 2009 and 0.028 in 2010 while hand pollinated plants had a higher S/O-ratio of 0.174. Thus, on average only about 2–5% of the ovules in a flower of *P. nudicaulis* will produce seed when open to pollinators.

## Discussion

Due to low pollinator abundance and the unreliability of pollinator services in an unfavorable and stochastic climate, plants that rely on pollinator services are considered to be extremely rare in arctic and alpine habitats. Indeed, our data indicates low visitation rates in both years and high variation in pollinator activity among years possibly due to differences in climate between the years. This pattern has been observed in other alpine tundra studies that correlate lower pollinator visits with reduced seed set [11], [14]. While data on visitation rates of arctic and subarctic alpine plants are not widely published, the mean visitation rate during periods of favorable weather (>10°C, light winds and not raining) for *Parrya nudicaulis* was 0.582 VFH in 2009 and 0.14 VFH in 2010. Visitation rates of *P. nudicaulis* correspond well to other alpine plant pollinator studies, but appear to be substantially lower compared to lower elevations and lower latitudes. Pollinator visitation rates in high elevation sites in Chile were 0.19 VFH [67]. The mean visitation rate of seven alpine species in Norway was 0.52 VFH in a favorable climate year [13]. Compared to other interior Alaskan plant species, syrphid fly visitation rates of 1.9 VFH was observed for flowers of *Saxifraga reflexa* in lowland bluff habitats [68]. Studies in temperate habitats generally range from approximately 1.8–3.5 VFH (for example [69]–[72]). Additional studies of pollinator visitation rates and pollinator efficiency are called for in the Arctic to confirm that visitation rates are indeed lower than in other biomes.

As observed in other arctic and alpine tundra pollinator studies [8], [9], [13], [15]–[17], the overwhelming majority of flower visitors to *P. nudicaulis* were dipterans. We observed that flies removed pollen from the tallest anthers at anthesis and rarely came in contact with the lower anthers. The lower anthers dehisce closer in time with stigma receptivity, but are well below the stigma which likely reduces auto-selfing. Pollinators of *P. nudicaulis* may be inefficient at transferring enough pollen between or within flowers. While others have observed pollinators to selectively forage on particular color morphs and contribute to color polymorphism maintenance within a population in other systems [50]–[53], we did not detect any significant visitation preference for flower color. Floral pigmentation may be largely influenced by indirect selection, such as drought tolerance, cold tolerance, and herbivory [73]. Indirect selection on flower color may be a stronger selective pressure in environments with extremely low pollinator visitation [74]. However, our sample size was low and more pollinator observations are necessary to confirm that pollinator preference for flower color is absent.

Supplemental hand pollination revealed that this population of *P. nudicaulis* was severely pollen limited. Hand pollination increased total seeds per plant by 500%. Comparatively, supplemental hand pollinations on average show a much lower (42%) increase in seeds per plant [4]. We also found the level of pollen limitation to be equal among the flower color variants of this species. The degree of pollen limitation is most severe in *P. nudicaulis* compared to other subarctic and arctic flowers with similar mating systems. Supplemental hand pollination on a subset of subarctic *Diapensia lapponica* flowers increased seed mass nearly 200% [75], a 47% increase of seed set was observed in arctic *Pedicularis lanata* [76], and a 72% increase of seed set in arctic *Saxifraga oppositifolia* [77]. Despite the predictions that pollen limitation should occur more frequently in habitats where pollinators are sparse, such as higher elevations and latitudes, a recent review [6] found this may not be the case. However, plant reproductive ecology and pollen limitation experiments manipulated at the whole inflorescence or plant level in alpine, subarctic, and arctic regions are scarce [6].

Seed set was surprisingly low in the open-pollination treatments in both years (2.37 seeds/plant in 2009 and 2.74 seeds/plant in 2010), given that we estimate *P. nudicaulis* to have approximately 140 ovules/plant on average. These two years contrasted dramatically in pollinator abundance during our presence and yet seed production per plant was similar suggesting that even in a stochastic environment, reproductive success was not stochastic. This is surprising given the high level of pollen limitation observed. It may be that in 2009 the more frequent observations of floral visitors were primarily ineffective pollinators and an uncommon pollinator that is less affected by inclement weather or active during non-observational hours may disproportionately impact pollen transfer.

High levels of pollen limitation are thought to promote the establishment of traits that increase auto-pollen deposition [21], [22]. A high level of reproductive assurance is believed to dominate plant mating systems of the arctic and subarctic floras [24]–[28] and furthermore, has been hypothesized to have evolved in response to the limited supply of pollinators [12], [23]. *Parrya nudicaulis* is in fact self-compatible, yet on average it produced only 0.45 seeds/plant in the absence of pollinators while pollinator presence increased seed production nearly five-fold. The cage treatment demonstrates that *P. nudicaulis* is mostly dependent on pollinators for seed set. The low level of auto-pollination is likely attributed to strong protandry, since spatial separation of anthers and stigma is minimal. Protandry greatly reduces self pollination and enhances outcrossing [78], but still carries the limitation of relying on pollinator services as well as not effectively reducing between-flower selfing (geitonogamy). However, geitonogamy is likely to be low due to flowers being asynchronous within the inflorescence where generally only two to four flowers are open at a time. Furthermore, while our pollinator data is limited, our observations suggest that most of the likely pollinators (syrphid, dance, and muscid flies) typically visit just one or two flowers before leaving the inflorescence, and therefore overall we expect geitonogamy to be minimal.

The P/O-ratio in this species is consistent with a facultatively outcrossing mating system. The only arctic and subarctic plants known to have a similar P/O-ratio are *Diapensia lapponica, Saxifraga oppositifolia* [79], *Pedicularis lanata*, *Pedicularis lapponica*, [76], and *Primula tschuktschorum* [29]. With a high P/O ratio, early flowering time, and a pollinator dependency system, the life history strategy of *P. nudicaulis* can be classified as a pollen-risk strategist that typically has a greater seed maturation time and outcrossing rate but lower seed set [79]. While P/O-ratios should be treated with some caution in characterizing mating system [80], genomic data from six genes across seven populations in Alaska (see [49]) resulted in inbreeding coefficients values consistent with a facultatively outcrossing mating system (*F*_IS_ = 0.60). Relative to 58 other species in the Brassicaceae from northern California [80], *P. nudicaulis* is intermediate in both ovules per flower and P/O-ratios. Preston [80] characterized these California taxa as either selfing or outcrossing and found very little overlap in P/O-ratios in these two mating categories. *Parrya nudicaulis*, however, has P/O-ratios that are at the maximum end of the range for the selfing taxa and the minimum end of the outcrossing taxa range. Additionally, the S/O-ratio in *P. nudicaulis* is much lower compared to other plant species. S/O-ratios are quite consistent within perennials, regardless of mating system, and on average have a S/O-ratio of 0.50 [66]. Even various arctic *Pedicularis* species have S/O-ratios that range from 0.29 to 0.56 [76]. Compared to other high latitude taxa and perennials in general, *P. nudicaulis* was found to have a much lower S/O-ratio of 0.02 in 2010 and 0.05 in 2009.

Plants may not benefit from extra pollen deposition since seed production can be constrained by other limiting factors such as soil resources, light, water, and climate [36]–[38], [40]. For example, *Ranunculis acris* increased seed set with supplemented pollen in lowland and artificially warmed alpine plants, suggesting that even with additional pollen, reproductive success in the alpine was constrained by temperatures [39], [40]. The Haig and Westoby [36] equilibrium model predicts that selection should favor traits that balance resources used to produce seed with pollinator attraction and pollen limitation should therefore be rare. Increased seed set from pollen addition may indicate that the population may not be in resource-pollination attraction equilibrium [1], [4] or it may indicate a “bet hedging” strategy is present in the mating system possibly due to a variance in the pollen deposition environment [4], [5], [46]. In contrast to the Haig and Westoby equilibrium, overproduction of ovules is predicted to be advantageous in stochastic environments where pollinators vary the mating success of flowers and flowers have increased fitness from chance pollination events [4], [5]. Several literature reviews support the idea that the overproduction of ovules is an adaptation to variable pollen receipt [1], [5], [46] and has been predicted to occur more frequently in higher elevations and variable climate habitats [5] like those of *P. nudicaulis*.

Pollinator visitation was insufficient in two years for complete seed set in *P. nudicaulis* at this population, with open pollinated flowers yielding only 0.33 seeds/fruit and hand pollinated flowers yielding 2.02 seeds/fruit, yet containing 11.60 ovules/fruit on average. There is a large gap in the potential female fitness suggesting resource limitation or an apparent overproduction of ovules. Seed set was not significantly different between the years in open pollinated plants despite contrasting climates and pollinator visitation rates suggesting that other abiotic resources may be stronger limiting factors of female fitness. While we did not explicitly test for a “bet hedging” strategy in *P. nudicaulis*, the apparent overproduction of ovules in this context suggests a few possible hypotheses: 1.) there is selection for increased male fitness through high pollen production, yet ovule and pollen production cannot respond separately to selection, 2.) a large number of zygotes die after fertilization, possibly due to lethal genetic traits in the population, 3.) plants are simultaneously bet-hedging on two separate limiting factors (pollen availability and abiotic resources).

We recommend future studies employ manipulative experiments on the limiting factors of resources and pollen availability and explicitly test the hypothesis of an overproduction of ovules as a bet hedging strategy in alpine and higher latitude habitats. To evaluate the magnitude of pollen limitation in the Arctic more generally, we envision assessing S/O-ratios of open and hand pollinated plants for populations along a latitudinal cline of wide ranging species, such as *P. nudicaulis*. Second, evolutionary responses to low pollinator visitation in the Arctic can be explored more broadly by comparisons of reproductive traits using phylogenetically explicit methods [81] for clades with sufficient resolution that have both arctic and lower latitude members. For example, hypotheses can be devised to test the Haig Westoby equilibrium such as, “is there evidence for the evolution of fewer ovules in Arctic species?” Evolutionary responses to selection based on the bet-hedging hypothesis, is not necessarily predicted to result in greater ovule number in regions of variable pollen receipt, however. Resolution of evolutionary relationships in *Parrya* is not sufficient for testing these ideas currently (see [82]), and ovule number per fruit does not show clear patterns with the four North American species (two of which are exclusively Arctic [83]), though species from China [84] appear to have higher ovule numbers than any of the North American species.

### Conclusions

Arctic and alpine pollinators are subject to stochastic weather and may limit the reproductive success of plants reliant on them in these regions. The pollinator assemblage of *P. nudicaulis* was dominated by flies and did not appear to favorably visit a particular color morph more frequently. The pollinating insect assembly may be inefficient at creating sufficient seed set as supplemental hand pollinations at the whole plant level demonstrated that this population is greatly limited by pollen. A limited supply of pollen has been hypothesized to be an evolutionary driving force for reproductive assurance in the arctic and alpine flora. In contrast, we have demonstrated that an arctic to subarctic taxon is highly dependent upon pollinators for seed set. Furthermore, the P/O-ratio of *P. nudicaulis* places it in the lower end of the outcrossing mating system spectrum compared to other Brassicaceae species but should still be considered facultative xenogamous. An evolutionary cause for pollen limitation in *P. nudicaulis* is possibly due to an over production of ovules as a “bet-hedging” strategy, which is predicted to be more common in environments with variable pollen receipt such as higher elevations. Additionally resource limitation may also contribute to the appearance of pollen limitation and therefore *P. nudicaulis* may be limited by both pollen and resources. Reproductive biology and experimental manipulations on pollen limitation at the whole plant level of higher latitude plant species are rarely studied, leaving gaps in our knowledge of potential evolutionary consequences of the plant-animal interaction dynamics in these regions. Selection pressures of pollinators on floral traits in such environments have yet to be explored. With a low fecundity, *P. nudicaulis* potentially faces difficulty in responding to the rapidly changing environment from climate change.
